# 23-Hydroxyursolic Acid Isolated from the Stem Bark of *Cussonia bancoensis* Induces Apoptosis through Fas/Caspase-8-Dependent Pathway in HL-60 Human Promyelocytic Leukemia Cells

**DOI:** 10.3390/molecules23123306

**Published:** 2018-12-13

**Authors:** Jong-Heon Won, Kyung-Sook Chung, Eun-Young Park, Jeong-Hun Lee, Jung-Hye Choi, Leon Azefack Tapondjou, Hee-Juhn Park, Masaaki Nomura, Ahmed H.E. Hassan, Kyung-Tae Lee

**Affiliations:** 1 Department of Pharmaceutical Biochemistry, College of Pharmacy, Kyung Hee University, Seoul 02447, Korea; ppoi788@gmail.com (J.-H.W.); adella76@hanmail.net (K.-S.C.); evolutioner@hanmail.net (E.-Y.P.); ztztzt08@hanmail.net (J.-H.L.); 2 Life and Nanopharmaceutical Science, College of Pharmacy, Kyung Hee University, Seoul 02447, Korea; jchoi@khu.ac.kr; 3 Oriental Pharmaceutical Science, College of Pharmacy, Kyung Hee University, Seoul 02447, Korea; 4 Department of Chemistry, Faculty of Science, University of Dschang, Box 183, Dschang, Cameroon; tapondjou2001@yahoo.fr; 5 Division of Applied Plant Sciences, Sang-Ji University, Wonju 220-702, Korea; hjpark@sangji.ac.kr; 6 Department of Clinical Pharmacology, Faculty of Pharmaceutical Sciences, Hokuriku University, Kanazawa, Ishikawa 920-1181, Japan; m-nomura@hokuriku-u.ac.jp; 7 Department of Pharmacy, College of Pharmacy, Kyung Hee University, Seoul 02447, Korea; ahmed_hassan@khu.ac.kr; 8 Department of Medicinal Chemistry, Faculty of Pharmacy, Mansoura University, Mansoura 35516, Egypt

**Keywords:** 23-hydroxyursolic acid, apoptosis, caspase, Bcl-2, mitochondria, death-inducing signaling complex

## Abstract

The natural product 23-hydroxyursolic acid (23-HUA) is a derivative of ursolic acid, which is known to induce cancer cell apoptosis. However, apoptotic effects and mechanisms of 23-HUA have not been well characterized yet. Herein, we investigated the molecular mechanisms of 23-HUA-induced apoptosis in HL-60 human promyelocytic leukemia cells. 23-HUA-treated HL-60 cells showed apoptotic features including internucleosomal DNA condensation and fragmentation as well as externalization of phosphatidylserine residues. 23-HUA induced a series of mitochondrial events including disruption of mitochondrial membrane potential (*ΔΨ_m_*), cytochrome *c* and Smac/DIABLO release and loss of balance between pro-apoptotic and anti-apoptotic Bcl-2 proteins in HL-60 cells. In addition, 23-HUA activated caspase-8, caspase-9 and caspase-3. Pretreatment with a broad caspase inhibitor (z-VAD-fmk), a caspase-3 inhibitor (z-DEVD-fmk), and a caspase-8 inhibitor (z-IETD-fmk) significantly attenuated 23-HUA-induced DNA fragmentation. After 23-HUA-induced apoptosis, proteins expression levels of FasL, Fas and FADD constituting the death-inducing signaling complex (DISC) were upregulated in HL-60 cells. Moreover, transfection with Fas or FADD siRNA significantly blocked 23-HUA-induced DNA fragmentation and caspases activation. Taken together, these findings indicate that 23-HUA induces apoptosis in HL-60 human promyelocytic leukemia cells through formation of DISC and caspase-8 activation leading to loss of *ΔΨ_m_* and caspase-3 activation.

## 1. Introduction

Apoptosis is a programmed cell death triggered by many chemotherapeutic agents within leukemic cells [1]. In fact, it is a highly organized cell death characterized by well-defined processes encompassing cell volume decrement, cytoplasmic organelles compaction, nuclear chromatin condensation and fragmentation, internucleosomal DNA cleavage, membrane blebbing and activation of a family of cysteinyl aspartate-specific proteinase called caspases [2]. Apoptosis anomalies are implicated in various diseases including cancer, neurodegenerative disorders and acquired immunodeficiency syndrome (AIDS) [3]. Depending on the type of the apoptotic stimulus, apoptosis might be triggered by death receptor-dependent extrinsic apoptotic pathway or mitochondria-dependent intrinsic pathway, which are the two major apoptosis pathways [4]. In the extrinsic apoptotic pathway, a death-inducing signaling complex (DISC) is formed by the interaction between Fas and Fas ligand (FasL). Consequently, Fas recruits adaptor molecules including Fas-associated death domain (FADD) and procaspase-8 to form DISC that induces autocatalytic cleavage of procaspase-8 leading to caspase-8 activation. In turn, DISC-activated caspase-8 cleaves Bid into a truncated Bid (tBid) that initiates the intrinsic apoptotic pathway resulting in the release of cytochrome *c* and Smac/DIABLO from the mitochondria and triggering effector caspase cascade activation including caspase-3 and caspase-7, then eventually, apoptosis [5].

**Figure 1:**
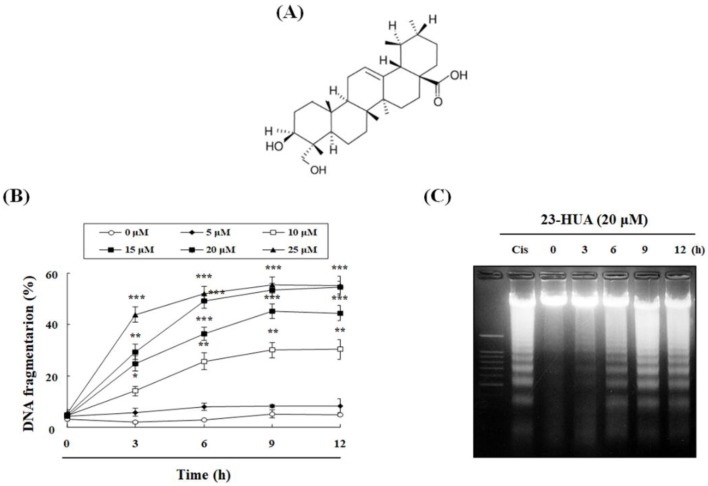

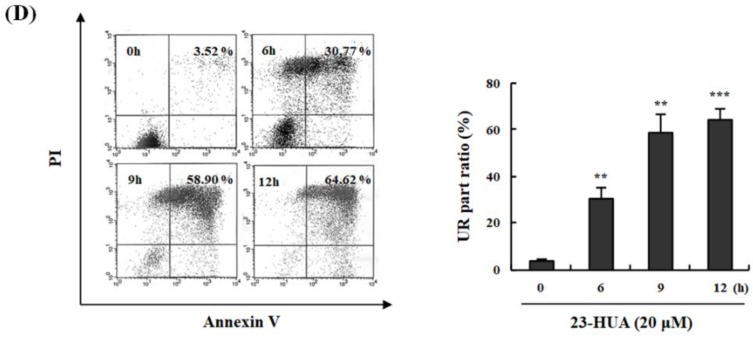
Induction of apoptosis in 23-HUA-treated HL-60 cells. (**A**) Chemical structure of 23-hydroxyursolic acid (23-HUA). (**B**) Percentages of DNA fragmentation was determined by fluorometric analysis using DAPI. (**C**) Fragmented DNA of HL-60 cells treated with 20 µM 23-HUA for 0, 3, 6, 9 and 12 h detected using 2% agarose gel after visualization with ethidium bromide stain. HL-60 cells treated with 50 µM cisplatin (Cis) were used as positive control. (**D**) Cells were co-stained with PI and FITC-conjugated annexin V after treatment with 20 µM 23-HUA to detect externalization of phosphatidylserine (PS) followed by flow cytometric analysis. Data are means ± S.D. of triplicate experiments. * *p* < 0.05, ** *p* < 0.01 and *** *p* < 0.001 vs. control group.

Triterpenoids are a large group of phytochemicals [6]. The exponential increase in bioactive triterpenoids reports over the last decade reflects their growing importance as sources of medications and preventive medicines [7]. Several studies have elucidated that various triterpenoid members elicit diverse bioactivities including anti-oxidant, anti-microbial, anti-viral, anti-allergic, anti-pruritic, anti-angiogenic and spasmolytic activities [8,9]. In addition, some triterpenoids have been found to elicit selective cytotoxicity against cancer cells rather than normal cells [10,11,12]. Among them, the ursane-type pentacyclic ursolic acid (3β-hydroxyurs-12-en-28-oic-acid, Figure 1) has been reported to elicit in vitro and in vivo bioactivity against cancer models [13,14,15]. Despite in vitro and in vivo studies provided evidences of the pivotal roles of ursolic in cancer, mechanism studies were little reported in cancer cells.

Previously, we have determined the cytotoxic potency and anti-tumor activity of some triterpenes against cancer cells [16,17]. In addition, we have isolated 23-hydroxyursolic acid (23-HUA, Figure 1A) from *Cussonia bancoensis* indigenous to dense humid forests extending from Ivory Coast to Nigeria [18]. We have also found that 23-HUA inhibited cell growth via induction of caspase-dependent apoptosis in human HeLa cells [19]. However, the underlying mechanisms responsible for the 23-HUA-induced apoptosis remains undefined. Therefore, we investigated molecular mechanism involved the cytotoxic properties of 23-HUA through the formation of DISC by Fas-FasL binding and activation of caspase cascade in HL-60 leukemia cells.

## 2. Results

### 2.1. 23-HUA Causes Apoptosis in HL-60 Cells

Initially, we have measured 23-HUA-induced cytotoxicity against eight different cancer cell lines using 3-(4,5-dimethylthiazol-2-yl)-2,5-diphenyltetrazolium bromide (MTT) assay. As shown in Table 1, 23-HUA has less cytotoxic effect on L132 normal cells compared with cancer cells. In addition, the cytotoxicity of 23-HUA was most prominent in HL-60 human promyelocytic leukemic cells, this cell line was selected for further investigations of 23-HUA-induced apoptosis. Because HL-60 cell line is also an attractive model for studies of differentiation which is induced by any agents within 24 h, we treated the higher concentration of 23-HUA (20 μM) than IC_50_ (11.07 μM) to optimize for detecting the 23-HUA-induced apoptosis in HL-60 cells. Consequently, we measured DNA fragmentation within HL-60 cells treated with increasing concentrations of 23-HUA (5, 10, 15, 20 or 25 µM) at various time intervals of 0, 3, 6, 9, and 12 h using 4′,6-diamidino-2-phenylindole (DAPI) staining. As Figure 1B illustrates, 23-HUA induced a time- and concentration-dependent increase in DNA fragmentation in HL-60 cells.

To confirm apoptosis but not necrosis as the type of 23-HUA-induced cell death, we investigated internucleosomal DNA fragmentation and performed propidium iodide (PI) and annexin V-FITC double staining at increasing time intervals. In agarose gel electrophoresis, DNA ladder as a hallmark of apoptosis-induced internuleosomal DNA fragmentation was induced by treatment with 20 µM 23-HUA in HL-60 cells in a time-dependent manner (Figure 1C). In addition, we examined whether 23-HUA could induce phosphatidylserine (PS) exposure in HL-60 cells employing biparametric cytofluorimetric analysis using PI and annexin V-FITC to stain DNA and PS, respectively. As shown in Figure 1D, 23-HUA treatment significantly increased the ratio of PI and annexin V-positive HL-60 cells in a time-dependent manner. Collectively, these results indicate that 23-HUA induces HL-60 leukemia cells death via apoptosis rather than non-specific necrosis.

### 2.2. 23-HUA Disrupts Mitochondrial Membrane Potential and Regulates Translocation of Mitochondria-Related Bcl-2 Family Proteins in HL-60 Cells

**Figure 2:**
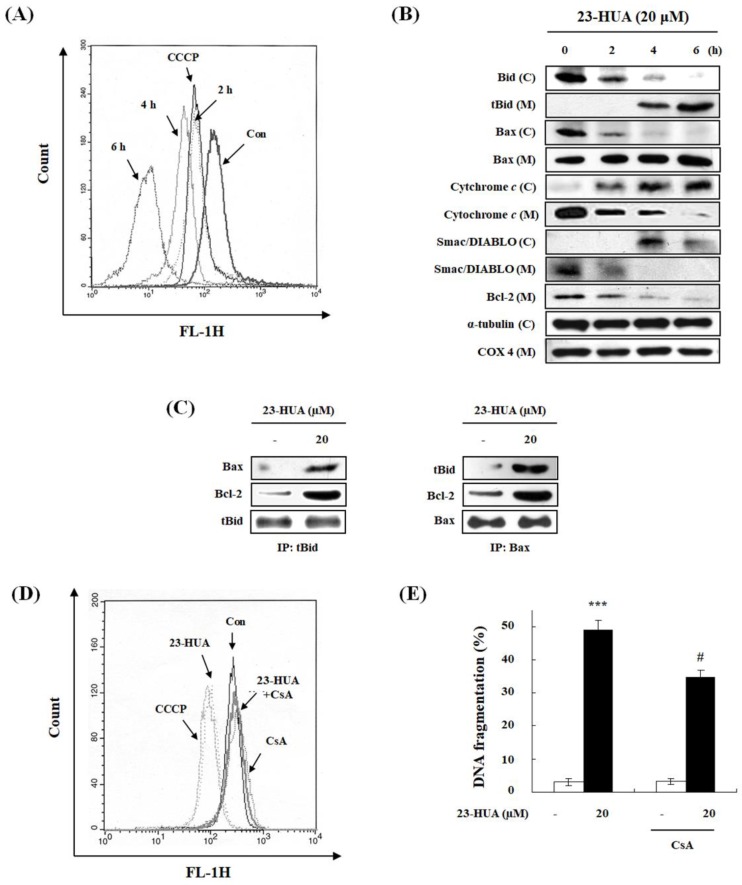
Dissipation of *ΔΨ_m_* and release of pro-apoptotic proteins from mitochondria following 23-HUA-caused apoptosis. (**A**) Analysis of *ΔΨ_m_* after treatment with 20 µM 23-HUA (0, 2, 4 and 6 h) using CCCP as a positive control. (**B**) Western blot analysis after treatment with 20 µM 23-HUA (0, 2, 4 and 6 h, C: cytosol, M: mitochondria). α-Tubulin and COX 4 were used as a positive control. (**C**) HL-60 cells were treated with or without 20 µM 23-HUA for 4 h. Cell lysates were immunoprecipitated with anti-tBid or anti-Bax antibody followed by western blot analysis using anti-tBid, anti Bax, and anti-Bcl-2 antibody (IP: immuonprecipitation). (**D**) HL-60 cells were pre-treated with 5 µM cyclosporin A (CsA) followed by treatment with 20 µM 23-HUA for additional 6 h. For analysis of *ΔΨ_m_*, cells were treated with 50 nM DiOC_6_ for 30 min. (**E**) DNA fragmentation was detected by DAPI assay. Values are the means ± SD of triplicate experiments. *** *p* < 0.001 vs. control group, # *p* <0.05 vs. 23-HUA-treated group.

Considering the crucial role of the mitochondrial pathway in apoptosis [20], we examined mitochondrial membrane potential (*ΔΨ_m_*) and Bcl-2 family proteins alterations in 23-HUA treated HL-60 cells. As shown in Figure 2A, treatment with either 23-HUA (20 µM) or the oxidative phosphorylation uncoupling agent carbonyl cyanide *m*-chlorophenylhydrazone (CCCP, 50 μM) resulted in a significant reduction of *ΔΨ_m_* time-dependently. To explore the underlying mechanism of 23-HUA-induced *ΔΨ_m_* change in HL-60 cells, we have examined the translocation of cytosolic tBid and Bax into mitochondria. As shown in Figure 2B, treatment of HL-60 cells with 23-HUA increased mitochondrial levels of pro-apoptotic Bax and tBid. The increased levels of pro-apoptotic proteins in mitochondria might trigger the release of cytochrome *c* and Smac/DIABLO from mitochondrial intermembrane spaces resulting in apoptosis [21]. To verify whether translocation of cytochrome *c* and Smac/DIABLO were involved in 23-HUA-induced apoptosis, we measured cytochrome *c* and Smac/DIABLO levels in cytosolic proteins by western blot analysis. Indeed, translocation of cytochrome *c* and Smac/DIABLO from mitochondria to cytosol was observed in 23-HUA-treated HL-60 cells. Furthermore, the anti-apoptotic Bcl-2 proteins levels were decreased following 23-HUA treatment. For further assessment of the role of Bcl-2 family proteins, we performed immunoprecipitation analysis to explore whether 23-HUA had an effect on the interactions between tBid, Bax and Bcl-2 in HL-60 cells (Figure 2C). After 4 h treatment with 23-HUA, tBid associations to Bax and Bcl-2 as well as Bax associations to tBid and Bcl-2 strongly increased relative to the untreated control cells. These results suggest that tBid and Bax could directly bind to Bcl-2 resulting in loss of the dimerization between anti-apoptotic Bcl-2 family proteins in 23-HUA-treated HL-60 cells. No significant changes in expression of other Bcl-2 family proteins (Bcl-xL, Bak and Bad) were observed in 23-HUA-treated HL-60 cells (data not shown). To examine the possible contribution of *ΔΨ_m_* reduction to 23-HUA-induced apoptosis, the potent permeability transition pore inhibitor CsA was used. Pretreatment with CsA completely recovered the loss of *ΔΨ_m_* (Figure 2D) and partially inhibited DNA fragmentation in 23-HUA-treated HL-60 cells (Figure 2E). Collectively, these results indicate that 23-HUA can induce *ΔΨ_m_* loss and alters expression levels of mitochondria-related proteins that appear to mediate the 23-HUA-stimulated apoptosis.

### 2.3. Caspase-Dependent Pathway Is Involved in 23-HUA-Induced Apoptosis

**Figure 3:**
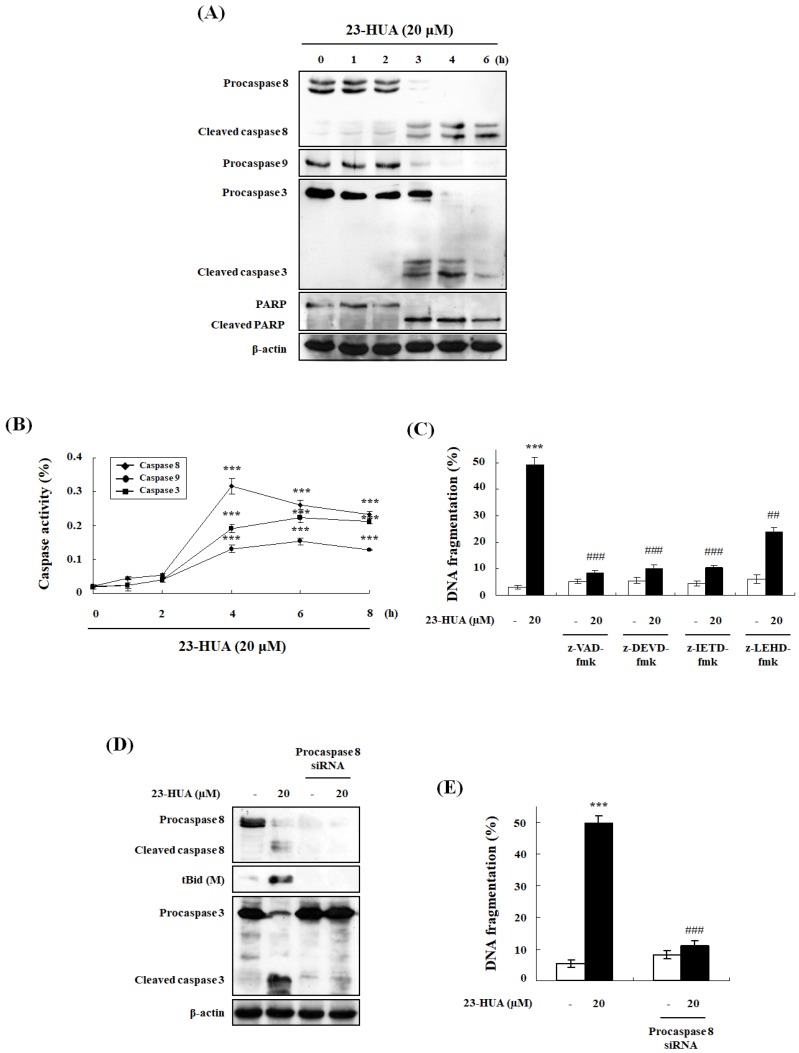
Involvement of caspase cascade in 23-HUA-induced apoptosis. (**A**) Proteins expression levels determined by western blot analysis of 20 µM 23-HUA-treated HL-60 cells (0, 1, 2, 3, 4 or 6 h). β-actin was used as an internal control. (**B**) Caspase-8, -9, and -3 activities were measured using colorimetric substrates Ac-IETD-pNA, Ac-LEHD-Pna and Ac-DEVD-pNA respectively. (**C**) HL-60 cells were pretreated with caspase inhibitors (50 μM z-VAD-fmk, z-DEVD-fmk, z-IETD-fmk, or z-LEHD-fmk) for 1 h followed by 20 µM 23-HUA treatment for 6 h. (**D**) HL-60 cells transfected with procaspase-8 siRNA were treated with 20 µM 23-HUA for 6 h. Proteins expression levels were determined by western blot analysis. (**E**) HL-60 cells transfected with procaspase-8 siRNA were treated with 20 µM 23-HUA for 6 h. 23-HUA-induced DNA fragmentation was determined by DAPI assay. Values are the means ± SD of triplicate experiments. *** *p* < 0.001 vs. control group, ## *p* < 0.01, ### *p* < 0.001 vs. 23-HUA-treated group.

To investigate the involvement of caspase activation in 23-HUA-induced apoptosis, HL-60 cells were treated with 20 µM 23-HUA and then caspases activation and cleavage of poly ADP-ribose polymerase (PARP, an endogenous caspase-3 substrate) were determined using western blot analysis. As shown in Figure 3A, 23-HUA time-dependently cleaved procaspase-8, -9 and -3 as well as PARP in HL-60 cells. Next, enzymatic activities of caspase-8, -9, and -3 in 23-HUA-treated cells were measured using specific colorimetric substrates. The results showed that 23-HUA increased the enzymatic activities of caspase-8, -9, and -3 in a time-dependent manner (Figure 3B). To determine the contribution of these caspases to the 23-HUA-induced apoptosis in HL-60 cells, z-VAD-fmk (a broad caspase inhibitor), z-DEVD-fmk (a caspase-3 inhibitor), z-IETD-fmk (a caspase-8 inhibitor), and z-LEHD-fmk (a caspase-9 inhibitor) were added to counteract caspases activations.

Not only z-VAD-fmk, but also z-DEVE-fmk and z-IETD-fmk completely blocked the 23-HUA-induced DNA fragmentation whereas z-LEHD-fmk partially inhibited 23-HUA-induced DNA fragmentation (Figure 3C). Caspase-8 is an initiator caspase that plays an essential role in other caspases activation including caspase-3 and -9 as well as the subsequent triggered apoptosis [22]. Therefore, we have silenced expression of caspase-8 to evaluate its impact on 23-HUA-induced procaspase-3 cleavage, truncated Bid and DNA-fragmentation. As shown in Figure 3D, caspase-8 levels were markedly downregulated in caspase-8 siRNA-transfected HL-60 cells relative to cells transfected with control siRNA. In addition, caspase-8 siRNA transfection significantly blocked 23-HUA-induced activation of the caspase-8, -3, cleavage of Bid and DNA fragmentation in HL-60 cells in comparison with control siRNA transfected cells (Figure 3D,E). Taken together, these findings confirm the crucial role of caspase-8 activation in 23-HUA-induced apoptosis.

### 2.4. 23-HUA-Induced Apoptosis Requires DISC Formation in HL-60 Cells

**Figure 4:**
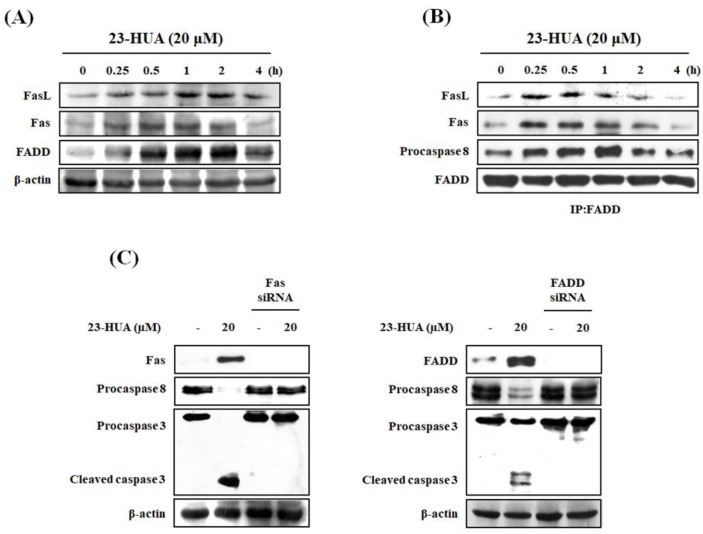

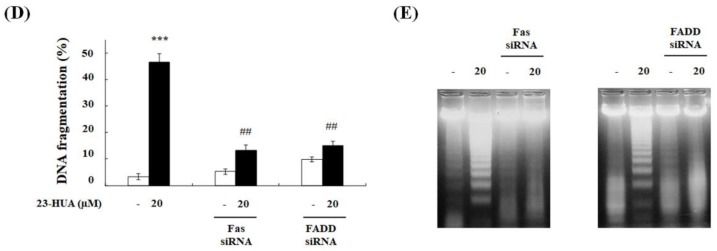
Effect of 23-HUA on Fas-related proteins expression in HL-60 cells. (**A**) Proteins expression levels were measured by western blot analysis in 20 µM 23-HUA-treated HL-60 cells (0, 0.25, 0.5, 1, 2 and 4 h). β-Actin was used as an internal control. (**B**) Cell lysates immunoprecipitation with anti-FADD antibody followed by western blot analysis (IP: immunoprecipitation). (**C**) HL-60 cells transfected with Fas or FAD siRNA were treated with 20 µM 23-HUA for 6 h. Proteins expression levels were measured by western blot analysis. β-Actin was used as an internal control. (**D**) HL-60 cells transfected with Fas or FADD siRNA were treated with 20 µM 23-HUA for 6 h. 23-HUA-induced DNA fragmentation was measured by DAPI assay. (**E**) Apoptotic DNA fragmentation was detected by 2% agarose gel electrophoresis and visualized by ethidium bromide stain. Values are the means ± SD of triplicate experiments. *** *p* < 0.001 vs. control group, ## *p* < 0.01 vs. 23-HUA-treated group.

Caspase-8 activation can be initiated through death receptor signaling [4]. Therefore, we examined FasL, Fas, and FADD proteins expression levels in 23-HUA-treated HL-60 cells that turned to be markedly increased. This suggested an enhanced FasL binding to its receptor Fas that is associated with the increased expression of these proteins in 23-HUA-treated HL-60 cells (Figure 4A). To determine the effect of 23-HUA on DISC formation, we performed immunoprecipitation using a FADD-specific antibody followed by immunoblotting with FasL, Fas, or procaspase-8 antibody. The results showed that interactions of FADD with FasL/Fas/procaspase-8 have increased at 0.25 h in 23-HUA-treated HL-60 cells (Figure 4B). To confirm the involvement of DISC in 23-HUA-induced apoptosis, we measured DNA fragmentation and caspase activation after Fas or FADD knockdown. As shown in Figure 4C, Fas and FADD levels were dramatically decreased in HL-60 cells transfected with Fas or FADD siRNA respectively relative to the control siRNA-transfected cells. In addition, 23-HUA failed to induce caspase-8 or caspase-3 activation in Fas or FADD siRNA-transfected cells. Moreover, 23-HUA-induced DNA fragmentation was markedly attenuated via Fas or FADD siRNA transfection (Figure 4D,E). These results suggest that 23-HUA may stimulate binding of FasL to its receptor Fas resulting in the formation of DISC comprising of FADD and procaspase-8 which subsequently and activates procasepase-8 by dimerization and autoproteolytic cleavage.

## 3. Discussion

Natural products have been used as folk medicines for several therapeutic purposes. In general, they are less toxic compared with synthetic chemical agents, thus providing safe sources of promising leads for the development of therapeutic drugs. This might explain the high interest in exploring phytochemicals as new effective and safe chemotherapeutic agents. Triterpenes constitute a major class of natural products. Several triterpenes were found to trigger cell death of various cancers [11]. Among them, pentacyclic triterpenes such as amooranin, asiatic acid, epi-oleanolic acid, oleanolic acid and ursolic acid suppress tumor-cell growth through apoptosis induction [6,23,24]. In this study, we used 23-HUA isolated from *Cussonia bancoensis* whose structure differs from asiatic acid only by the absence of the OH-group at position C-2. Nevertheless, MTT cytotoxicity assay showed that 23-HUA is more sensitive than asiatic acid on HL-60 (IC_50_; 11.07 vs. 46.67 μM) and HepG2 (IC_50_; 22.20 vs. 32.0 μM) cells [25,26]. Therefore, we speculate that C-2 might be an active group of triterpene acids which could strengthen cytotoxic activity.

Our results revealed that 23-HUA induced apoptosis, displaying fragmented DNA and externalization of PS in HL-60 cells. Apoptosis could be triggered in response to developmental cues or cellular stress [27]. It features morphological changes such as nuclear condensation and fragmentation, PS flipping and formation of apoptotic bodies [28]. The morphological characteristics of apoptosis result from cellular degradation triggered by cysteine proteases called caspases that cleave substrates after specific aspartate residues. Caspases exist as zymogens that are activated in response to apoptotic signals [5]. PARP is a nuclear enzyme that catalyzes the synthesis of poly(ADP-ribose) from nicotinamide adenine dinucleotide (NAD^+^), which is important for maintaining cells viability [29]. PARP is a major cleavage target of caspase-3 that facilitates cellular disassembly upon cleavage and serves as an indicator of cells undergoing apoptosis [30]. In our study, 23-HUA increased proteins levels of cleaved caspase-8, -9, -3 and PARP indicating caspases activation as one of the main pathways of the 23-HUA induced apoptosis in HL-60 leukemia cells. Pretreatment with z-VAD-fmk (a broad caspase inhibitor), z-IETD-fmk (a specific caspase-8 inhibitor), or z-DEVD-fmk (a specific caspase-3 inhibitor) significantly suppressed cells against 23-HUA-induced DNA fragmentation, whereas z-LEHD-fmk (caspase-9 inhibitor) partially protected cells against 23-HUA-induced apoptosis. Our results reveal that the 23-HUA-induced apoptosis involves caspase-8 and caspase-3-mediated mechanisms as the intrinsic mitochondrial pathway is insufficient to solely trigger the 23-HUA-induced apoptosis in HL-60 cells.

In the intrinsic apoptosis mode, the release of cytochrome *c* from mitochondria results in apoptosome formation that activates caspase-9 [31]. Mitochondria are essential as they generate ATP to supply cells with energy. They are also involved in the intrinsic apoptosis pathway as they release soluble proteins including cytochrome *c* and Smac/DIABLO from the intermembrane space to initiate caspase activation in the cytosol [32]. Herein, the results indicated that 23-HUA increased the translocation of cytochrome *c* from mitochondria, which in turn resulted in caspase-9 activation leading to activation of executioner caspases. In addition, 23-HUA also increases the release of Smac/DIABLO into the cytoplasm. These findings indicate that translocation of cytochrome *c* and Smac/DIABLO from mitochondria into the cytoplasm might contribute to the 23-HUA-induced apoptosis in HL-60 human leukemia cells. The translocation of mitochondria-mediated proteins is a consequence of a compromised mitochondrial outer membrane integrity known as mitochondrial outer membrane permeabilization that is controlled by oligomer formation within pro-apoptotic Bcl-2 family members [33]. Bcl-2 family proteins are divided into anti- and pro-apoptotic groups participating in apoptosis inhibition or activation, respectively [32]. Anti-apoptotic proteins such as Bcl-xL, Bcl-2 and Mcl-1 inhibit oligomerization of pro-apoptotic proteins including Bax and Bak leading to permeabilized mitochondria. However, BH3-only proteins can directly (Bid and Bim) or indirectly (Bad) activate Bax and Bak [34]. Our findings revealed that 23-HUA increased mitochondrial translocation and proteins interaction of tBid and Bax, suggesting conformational changes and intermolecular contacts of Bid at the initiation of apoptosis. Bid is truncated by activated caspase-8, resulting in generation of a 15 kDa protein called tBid that plays as a messenger between death receptor and mitochondria to initiate apoptosis [5].

**Figure 5:**
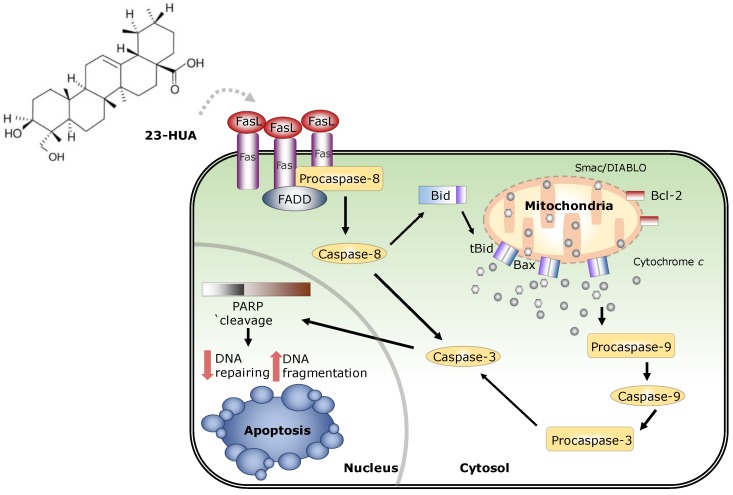
23-hydroxyursolic acid-induced apoptosis via Fas/caspase-8-dependent pathway in HL-60 human promyelocytic leukemia cells.

Caspase-8 activation is triggered by clustering of death domain and death receptors. Binding of FasL to Fas leads to receptor trimerization and formation of DISC. Adaptor protein FADD is recruited by DISC where the death domains of both proteins can interact with clustered caspases-8 which in turn results in self-activation via a cross-proteolysis mechanism [31]. Consistent with this, 23-HUA increased the interaction of DISC formation protein. In addition, 23-HUA-induced DNA fragmentation was diminished after the knockdown of Fas or FADD in HL-60 cells. As a whole, our results suggest that 23-HUA-induced apoptosis involves DISC-associated caspase-8 activation that leads to cleavage of Bid, which is a bridging element between the extrinsic death receptor pathway and the intrinsic mitochondrial pathway (Figure 5). Taken together, our finding provides evidences suggesting 23-HUA as a potential therapeutic agent for treatment of human promyeliocytic leukemia.

## 4. Materials and Methods

### 4.1. Materials

23-HUA was isolated from *C. bancoensis* and its structure was confirmed spectroscopically (^1^H-NMR, ^13^C-NMR, IR and MS) as reported previously [18] The purity of the isolated 23-HUA was determined to be >97% (HPLC). MTT, β-actin antibody, protein A, DAPI, PI and all other chemicals were purchased from Sigma Chemical Co. (St. Louis, MO, USA). Primary antibodies targeting cytochrome *c*, caspase-8, FasL and FADD were obtained from BD Biosciences Pharmingen (San Jose, CA, USA). Antibodies for caspase-3, caspase-9, Bid, tBid, Bcl-2, Bax, PARP, Fas, Smac/DIABLO, α-tubulin, COX 4 and secondary antibody were purchased from Santa Cruz Biotechnology, Inc (Santa Cruz, CA, USA). Caspase inhibitors (z-VAD-fmk, z-DEVD-fmk, z-IETD-fmk, and z-LEHD-fmk) were purchased from Calbiochem (San Diego, CA, USA). siRNA for caspase-8, Fas, FADD and transfection reagents were purchased from Qiagen GmbH (Hilden, Germany).

### 4.2. Cell Cultures

HL-60 human promyelocytic leukemia, U937 human histocytic lymphoma, HeLa human negroid cervix epitheloid carcinoma, HepG2 human hepatoblastoma, P388D1 mouse lymphoblast, A172 human glioblastoma, A431 human epidermoid carcinoma and A549 human lung adenocarcinoma and L132 human lung epitherial cells were obtained from the Korean cell line bank (Seoul, Korea) and cultured in RPMI 1640 or Dulbecco’s modified Eagle’s minimum essential medium (DMEM) supplemented with 10% heat-inactivated fetal bovine serum (FBS), penicillin (100 units/mL), and streptomycin sulfate (100 µg/mL). Cells were maintained at 37 °C under a humidified atmosphere of 5% CO_2_.

### 4.3. MTT Assay

For cell viability determination, MTT assay was conducted according to the modified method reported by Lee et al. [35]. Briefly, cells were harvested while in the logarithmic growth phase, seeded in 96-well plates at a density of 2 × 10^4^ with a final volume of 190 μL/well then incubated for 24 h. 23-HUA (10 μL, full-range concentration) was added and cells incubated for 48 h then 50 μL/well of MTT (5 mg/mL stock solution in PBS) was added and incubated for 4 h. The supernatant was removed and MTT crystals were solubilized with 100 μL anhydrous DMSO. The optical density was measured at a wavelength of 540 nm.

### 4.4. DNA Fragmentation Assay

DNA fragmentation was quantified as previously reported [36].

### 4.5. PI and Annexin V Double Staining

Cells were harvested and washed twice with ice-cold PBS then resuspended with binding buffer (10 mM HEPES, pH 7.4, 140 mM NaCl, 2.5 mM CaCl_2_). The cell suspension (100 μL) was incubated in a dark place with 5 μL of annexin V-FITC and 10 μl of PI (50 μg/mL) for 15 min. Cells were analyzed for PI-annexin V double staining using FACS flow cytometry (Becton Dickinson Co, Heidelberg, Germany).

### 4.6. Determination of Mitochondria Membrane Potential (ΔΨ_m_)

Cells were collected, washed twice with ice-cold PBS, treated with 50 nM DiOC_6_ for 30 min and washed with PBS followed by FACS flow cytometric analysis. To ensure that DiOC_6_ uptake was specific for *ΔΨ_m_*, CCCP (50 μM) was used as a positive control. The potent mitochondrial permeability transition pore inhibitor cyclosporine A (CsA) was used to inhibit mitochondria membrane potential loss.

### 4.7. Preparation of Cytosolic and Mitochondrial Fractions

23-HUA-treated cells were extracted using mitochondrial fraction buffer (Active Motif, Brussels, Belgium) according to recommendations of the manufacturer. Cytosolic and mitochondrial fractions were then frozen in aliquots at −70 °C until required.

### 4.8. Western Blot and Immunoprecipitation Assay

For total cell protein extracts, cells were lysed in protein lysis buffer (PRO-PREP™, Intron Biotechnology, Seongnam, Korea). Protein concentration was determined using Bio-Rad protein assay agent (Hercules, CA, USA). The immunoprecipitation assay was conducted according to the reported method by Lee et al. [35]. Immunoblots were visualized by ECL kit (Amersham, Piscataway, NJ, USA).

### 4.9. Determination of Caspase Activity

The caspases activities were measured according to the method reported by Park et al. [36]. The optical density of the reaction mixture was quantified spectrophotometrically at a wavelength of 405 nm.

### 4.10. Gene Knockdown Using siRNA

Transfections were performed with siRNA (200 nM) by RNAiFect transfection reagent (Qiagen GmbH) according to recommendations of the manufacturer. After transfection, cells were incubated for 48 h prior to 23-HUA treatment.

### 4.11. Statistical Analysis

Data are expressed as mean ± S.D. Statistically significant values were compared using one-way ANOVA and Dunnett’s post hoc test using GraphPad Prism 5 statistical software (GraphPad Software Inc., San Diego, CA, USA). *p*-values less than 0.05 were considered statistically significant.

## Figures and Tables

**Table 1:** Effect of 23-HUA on cell growth against various cancer cell lines.

Cell Line	Origin	IC_50_ (μM) ^a^
HL-60	Human promyelocytic leukemia	11.07 ± 1.29
U937	Human histiocytic lymphoma	12.13 ± 1.55
HeLa	Human negroid cervix epitheloid carcinoma	16.16 ± 1.77
HepG2	Human hepatoblastoma	22.20 ± 1.07
P388D1	Mouse lymphoblast	13.42 ± 1.13
A172	Human glioblastoma	21.02 ± 1.14
A431	Human epidermoid carcinoma	24.93 ± 2.92
A549	Human lung adenocarcinoma	20.94 ± 2.37
L132	Human lung epithelial cell	32.57 ± 2.91

^a^ IC_50_ is the concentration that results in 50% reduction of cell viability relative to the control. Values are the means of a triplicate assay.
